# Highly sensitive *MLH1* methylation analysis in blood identifies a cancer patient with low-level mosaic *MLH1* epimutation

**DOI:** 10.1186/s13148-019-0762-6

**Published:** 2019-11-28

**Authors:** Estela Dámaso, Júlia Canet-Hermida, Gardenia Vargas-Parra, Àngela Velasco, Fátima Marín, Esther Darder, Jesús del Valle, Anna Fernández, Àngel Izquierdo, Gemma Mateu, Glòria Oliveras, Carmen Escribano, Virgínia Piñol, Hugo-Ikuo Uchima, José Luis Soto, Megan Hitchins, Ramon Farrés, Conxi Lázaro, Bernat Queralt, Joan Brunet, Gabriel Capellá, Marta Pineda

**Affiliations:** 1 Hereditary Cancer Program, Catalan Institute of Oncology, Institut d’Investigació Biomèdica de Bellvitge (IDIBELL), ONCOBELL Program, Av. Gran Via de l’Hospitalet, 199-203, 08908 L’ Hospitalet de Llobregat, Barcelona, Spain; 2 Department of General and Digestive Surgery, Dr Josep Trueta University Hospital, Girona, Spain; 3 Hereditary Cancer Program, Catalan Institute of Oncology, Institut d’Investigació Biomèdica de Girona (IDIBGI), Girona, Spain; 4 Pathology Department, Dr Josep Trueta University Hospital, Girona, Spain; 5 Pathology Department, Clínica Girona, Girona, Spain; 6 Department of Gastroenterology, Dr Josep Trueta University Hospital, Girona, Spain; 7 Hereditary Cancer Program Valencian Region, Molecular Genetics Laboratory, Elche University Hospital, Elche, Alicante, Spain; 8 Department of Biomedical Sciences, Cedars-Sinai Medical Center, CA, Los Angeles USA; 9 Centro de Investigación Biomédica en Red de Cáncer (CIBERONC), Madrid, Spain; 10 Department of Medical Oncology, Catalan Institute of Oncology, Girona, Spain; 11 Department of Medical Sciences Department, School of Medicine, University of Girona, Girona, Spain

**Keywords:** Constitutional *MLH1* epimutation, Lynch syndrome, Methylation, Epigenetic mosaicism, Highly sensitive methodologies

## Abstract

Constitutional *MLH1* methylation (epimutation) is a rare cause of Lynch syndrome. Low-level methylation (≤ 10%) has occasionally been described. This study aimed to identify low-level constitutional *MLH1* epimutations and determine its causal role in patients with *MLH1*-hypermethylated colorectal cancer.

Eighteen patients with *MLH1-*hypermethylated colorectal tumors in whom *MLH1* methylation was previously undetected in blood by methylation-specific multiplex ligation-dependent probe amplification (MS-MLPA) were screened for *MLH1* methylation using highly sensitive MS-melting curve analysis (MS-MCA). Constitutional methylation was characterized by different approaches.

MS-MCA identified one patient (5.6%) with low-level *MLH1* methylation (~ 1%) in blood and other normal tissues, which was confirmed by clonal bisulfite sequencing in blood. The patient had developed three clonally related gastrointestinal *MLH1*-methylated tumor lesions at 22, 24, and 25 years of age. The methylated region in normal tissues overlapped with that reported for other carriers of constitutional *MLH1* epimutations. Low-level *MLH1* methylation and reduced allelic expression were linked to the same genetic haplotype, whereas the opposite allele was lost in patient’s tumors. Mutation screening of *MLH1* and other hereditary cancer genes was negative.

Herein, a highly sensitive MS-MCA-based approach has demonstrated its utility for the identification of low-level constitutional *MLH1* epigenetic mosaicism. The eventual identification and characterization of additional cases will be critical to ascertain the cancer risks associated with constitutional *MLH1 *epigenetic mosaicism.

## Introduction

Recent findings have indicated that underlying epimutations of certain genes in the normal tissue are associated with an elevated risk of particular tumor types [1–5]. This indicates that epigenetic events, apart from genetic alterations, maybe the initial step in the carcinogenesis process for these tumors [6]. When epimutations predisposing to disease are widely distributed across normal tissues, they are called constitutional epimutations.

Lynch syndrome (LS) is characterized by an increased risk for colorectal cancer (CRC) as well as other cancers (stomach, small intestine, and endometrium among others) [7]. It is mainly caused by germline genetic mutations in a mismatch repair (MMR) gene (*MLH1*, *MSH2*, *MSH6*, or *PSM2*). In a small proportion of patients, LS is caused by a *MLH1* constitutional epimutation, in which monoallelic hypermethylation of the promoter CpG island throughout normal tissues is linked to a constitutional allele-specific silencing [5].

Ninety-seven index cases with a constitutional *MLH1* epimutation have been reported so far [8–12]. Most are considered primary, arising apparently de novo and reversible between generations, whereas secondary epimutations are associated with a genetic variant in cis. Recently, we demonstrated that *EPM2AIP1-MLH1* CpG island is the sole differentially methylated region in primary *MLH1* epimutation carriers [9]. Available evidence suggests that constitutional epimutations cause a severe LS phenotype, including early-onset and multiple primary tumors [5].

The level of constitutional methylation varies among *MLH1* epimutation carriers. Although the majority of cases identified to date have shown hemiallelic *MLH1* methylation in blood, variable levels of methylation have frequently been reported (reviewed in [9]), including seven cases harboring low methylation levels (≤ 10%) [13–16]. Nevertheless, low-level *MLH1* methylation in blood has also been reported in healthy controls, confounding its interpretation [17]. Thus, the use of robust and sensitive approaches is critical to determine the true prevalence of constitutional epimutations and to ascertain a putative role of mosaic *MLH1* epimutation in cancer predisposition.

The main aim of this study was to identify patients with low levels of constitutional epigenetic mosaicism of the *MLH1* gene using highly sensitive methylation analysis techniques and explore its role in cancer predisposition.

## Patients and methods

### Patients and samples

Patients were identified through the Cancer Genetic Counseling Units at the Catalan Institute of Oncology from 1998 to 2016. Eighteen individuals presenting with *MLH1*-methylated CRC before 50 years of age, or multiple tumors before 60 years, were studied (Additional file 1: Figure S1; Additional file 2: Table S1) after excluding two previously reported bona fide constitutional *MLH1* epimutations [18]. Their levels of *MLH1* methylation in PBL (peripheral blood leukocytes) previously assessed by MS-MLPA were between 0 and 4% at the Deng C and D regions of the *MLH1* promoter CpG island, hence were below the limit of detection by this technique (10%) and considered negative (Additional file 2: Table S1) [19]. Twenty case-matched healthy individuals (matched by age, race, and geographic location) were included as controls. In addition, 61 LS cases harboring MMR genetic mutations, 12 constitutional *MLH1* epimutation carriers, and 41 healthy controls were included as reference groups for comparative global methylome analyses [9]. Written informed consent was obtained from all individuals, and the ethics committee of the respective hospitals approved the study. Sample processing is detailed in Additional file 3: Supplementary Methods.

### Methylation testing

The levels of methylation at the *MLH1* promoter in biological samples were assessed by several methods (Additional file 3: Supplementary Methods and Additional file 12: Table S5): (i) Methylation-specific multiplex ligation-dependent probe amplification (MS-MLPA) using the SALSA MLPA ME011 Mismatch Repair genes probemix (MRC-Holland). (ii) Methylation-specific melting curve analysis (MS-MCA): bisulfite-treated DNA was amplified within the Deng C and D regions in a nested PCR on a LightCycler 480 II; the analytical sensitivity of MS-MCA was assessed using serial dilutions (100, 75, 50, 25, 10, 5, 4, 3, 2, and 1%) of the RKO cell line (biallelic *MLH1* methylation; 100% methylated) into unmethylated (0% methylated) Whole Genome Amplification (WGA) DNA. The analytical sensitivity was 1% and 10% for the C and D regions, respectively (Additional file 4: Figure S2A-B). (iii) Pyrosequencing: bisulfite-treated DNA was amplified within the Deng C and intron 1 regions with biotin-labeled primers; the estimated analytical sensitivity was 4% and 5% at C-region and intron 1, respectively (Additional file 5: Figure S3A-B). (iv) Clonal bisulfite sequencing of fragments of the *MLH1* promoter encompassing the c.-93G>A promoter SNP was performed to confirm the low-level methylation detected by MS-MCA. (v) Finally, genome-wide methylation profiling was performed using Infinium Human Methylation 450K Beadchip, as previously described [9].

### *MLH1* expression and loss of heterozygosity analyses

Human lymphocytes from case 29 were cultured in PB-MAX Karyotyping Medium (Life Technologies, Carlsbad, CA) in the absence and presence of puromycin (Sigma, St. Louis, MO). Puromycin was used to prevent potential degradation of unstable transcripts by the nonsense-mediated decay mechanism. The impact of *MLH1* promoter methylation on allelic expression was assessed by measuring the relative levels of the two *MLH1* alleles at exonic SNP c.655A>G (rs1799977) in cDNA/gDNA by single-nucleotide primer extension (SNuPE), as previously described [18].

Loss of heterozygosity (LOH) was assessed by SNUPE as the ratio of tumor-DNA/distal normal-DNA at c.655A>G. Clonal sequencing of *MLH1* cDNA was performed to determine the phase between the heterozygous c.655A>G (exonic) and c.-93G>A (promoter) variants.

### Germline mutational analysis

Hereditary cancer genes (including *MLH1* gene) were screened for rare and mosaic germline variants, using analytical pipelines to detect mosaicism, as described in Additional file 3: Supplementary Methods.

### Immunohistochemical staining analysis

Formalin-fixed, paraffin-embedded tissue sections representative of the tumors were studied for CK7, CK20, CDX2, MUC1, MUC2, and MUC5 protein expression using standard immunohistochemistry techniques (see Additional file 3: Supplementary Methods).

### Somatic mutational analyses

Mutations in *KRAS*, *NRAS*, and *BRAF* were analyzed in tumors with the Idylla™ platform (Biocartis, Mechelen, Belgium). FFPE tumor tissue sections were placed directly into the Idylla system cartridge according to the manufacturer’s instructions (Idylla™ KRAS Mutation Test and NRAS-BRAF Mutation Test) and were analyzed for mutations in codons 12, 13, 59, 61, 117, and 146 of *KRAS*; in codons 12, 13, 59, 61, 117, and 146 of *NRAS*; and in codon 600 of *BRAF*.

Tumor samples were further analyzed using the NGS customized panel of 126 genes (I2HCP v2.1) as described above. To determine somatic variants, germline variants identified in the paired blood with a variant allele frequency (VAF) > 0.1 were subtracted from tumor variants. Variant calls with lower than 30x coverage, VAF < 0.05, or out of the region of interest (gene exons ± 20 bp) were excluded. Somatic variants with VAF > 0.2 in at least one tumor location were considered. The clonal relatedness between pairs of tumor samples based on their mutational profiles was tested using the SNVtest of the Clonality R package, which evaluates evidence for clonality against null hypothesis that the two tumors are independent [20]. Reference frequencies of somatic mutations were obtained from TCGA CRC MSI-H cohort with mutational data available (*n* = 28) [21]. For mutations not previously observed in the TCGA cohort, the reference frequency was set to 0.033 (1/29, being 29 the sum of the TCGA CRC MSI-H cases plus our patient).

## Results

### Highly sensitive *MLH1* methylation screening

**Fig. 1:**
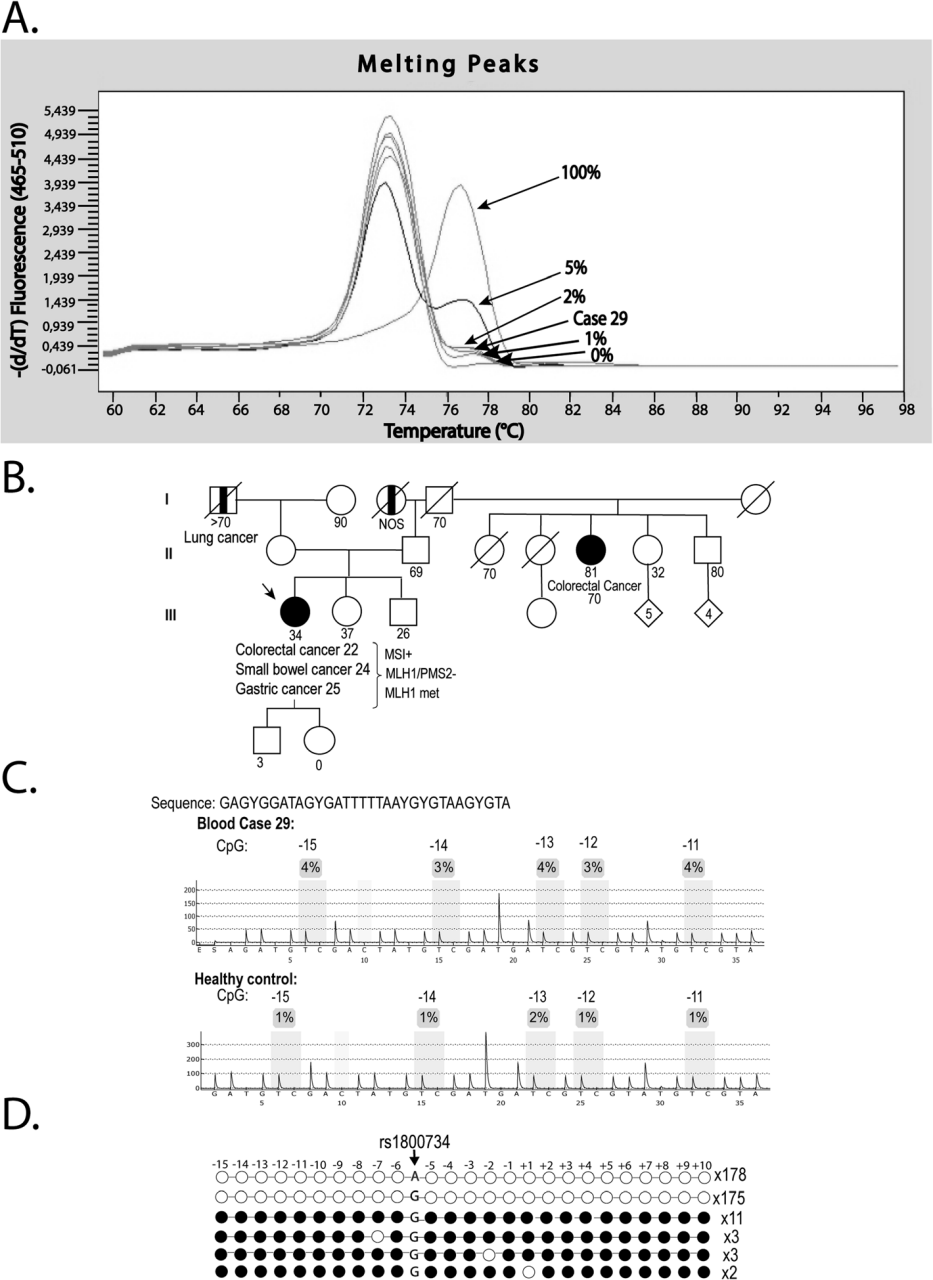
Identification and characterization of constitutional *MLH1* epigenetic mosaicism in case 29. **a** Methylation analysis by MS-MCA of the *MLH1* promoter C-region in blood DNA from case 29. The patient shows levels of methylation around 1%. **b** Family pedigree from case 29. The epimutation carrier is indicated by an arrow. Circles, females; squares, males; filled, cancer affected; vertical line at center, not otherwise specified. Cancer location and age at diagnosis are indicated. Generations are indicated on the left margin in Roman numerals. **c** Methylation analysis by pyosequencing of the *MLH1* promoter C-region in blood DNA from case 29 and one healthy control. Each CpG is numbered according its position relative to the translation initiation codon. **d** Clonal bisulfite sequencing of the *MLH1* promoter in PBL DNA from case 29. Each horizontal line represents a specific allele. CpG dinucleotides are depicted by circles. Black and white circles indicate methylated and unmethylated CpG sites, respectively. The allele at rs1800734 (c.-93G>A) is indicated as A or G. Methylation in case 29 is confined to the G allele. Each CpG analyzed is numbered according to its position relative to the translation initiation codon

The MS-MCA pattern of *MLH1* promoter (region C) of PBL from DNA healthy controls was the same as the unmethylated WGA sample, indicating the absence of detectable methylation in the control group (Additional file 4: Figure S2C). Likewise, 17 out of 18 patients harboring *MLH1*-hypermethylated CRC shared a non-methylated pattern. In contrast, PBL from case 29 showed the presence of methylation at a level of around 1% (Fig. 1a and Additional file 4: Figure S2D). After confirmation of the same low methylation levels in an independently extracted blood sample (Fig. 1a), we decided to study the case in depth.

### Clinicopathological characterization

Patient 29 is a woman who consecutively presented three *MLH1*-methylated gastrointestinal tumor lesions described as a low-grade (moderately differentiated) colorectal adenocarcinoma (pT4N1) at age 22, a well-differentiated small bowel adenocarcinoma (pT4N1) at age 24, and a well-differentiated tubular adenocarcinoma of the stomach (pT2N1) at 25 years of age (Fig. 1b). According to the clinical presentation and macroscopic description, the three lesions were treated as primary independent tumors. The patient has no disease recurrence after 9 years of follow-up. No family history of cancer was reported among her first-degree relatives (Fig. 1b).

**Fig. 2:**
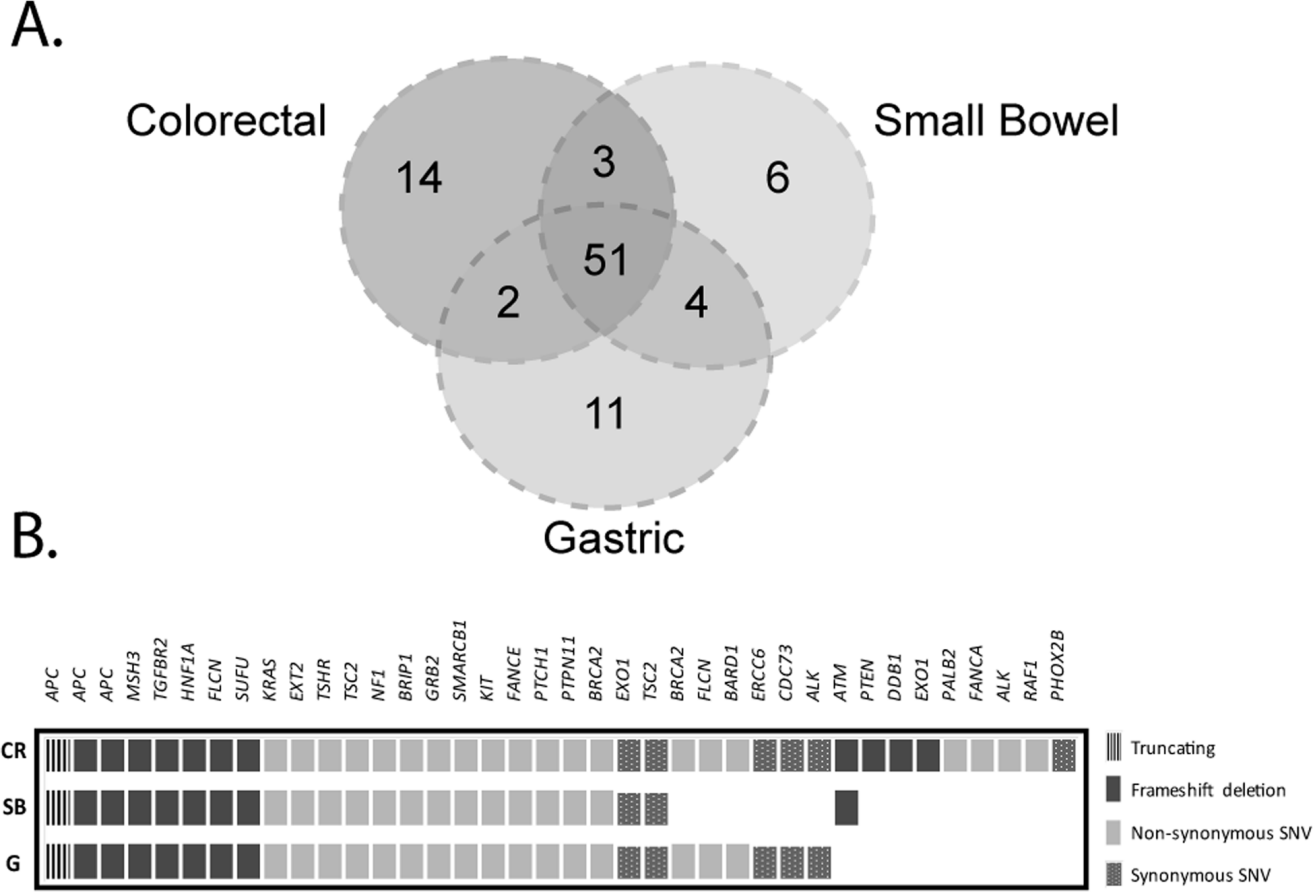
Somatic mutation profile of the different tumor lesions. **a** Venn diagram representing the total number of somatic mutations that are unique to each tumor lesion or shared at least between two tumor lesions. **b** Type of somatic mutations identified in each tumor sample. Only coding mutations have been considered. CR, colorectal; SB, small bowel; G, gastric

Of note, the three tumor lesions shared the same immunohistochemical staining pattern, showing negative expression of CK7 and CK20 markers and positive expression of CDX2, MUC1, MUC2, and MUC3 (Additional file 6: Figure S4). Also, 56% (51 out of 91) of the somatic variants identified in these lesions were shared between them, strongly suggesting a common origin (*p* < 0.001, clonality package) (Fig. 2a, b). The shared variants included mutations in key genes of intestinal carcinogenesis (e.g., *APC* K562fs, V782fs, and Q205X), well-known cancer driver mutations (*KRAS* G12D), and recurrent homopolymer deletions characteristic of MSI tumors (e.g., *TGFBR2* K153fs).

### Confirmation of the mosaic constitutional *MLH1* epimutation

The presence of *MLH1* methylation was confirmed by MS-MCA in all embryonic layers since methylation was detected in endodermal (gastric, small bowel, and colon mucosa) and ectodermal tissues (oral mucosa and skin fibroblasts) at similar levels than in PBL (mesoderm) (Additional file 4: Figure S2). In addition, slightly higher levels of methylation were also detected in normal tissue samples by pyrosequencing, but below the analytical sensitivity threshold of this technique (Fig. 1c; Additional file 5: Figure S3). In contrast, previous MS-MLPA analyses had reported only background methylation levels in blood DNA and in normal gastrointestinal tissues (≤ 10%) (Additional file 7: Table S2).

Considering the previous observations, *MLH1* methylation in blood was further assessed by clonal bisulfite sequencing (Fig. 1d) to confirm the presence and density of methylation in individual alleles. Nineteen methylated clones were identified out of 372 analyzed (5% methylated alleles; 95% CI 0.01–0.05), all of them displaying dense monoallelic methylation linked to the G allele at c.-93G>A (rs1800734), for which patient 29 was heterozygous. Of note, *in cis* genetic variants on the methylated G alleles were not detected in the region analyzed, including c.-27C>A (which has previously been linked to secondary *MLH1* epimutations) [22]. In all, clonal bisulfite sequencing supported the robustness of the MS-MCA results.

### Characterization and classification of *MLH1* epimutation

**Fig. 3:**
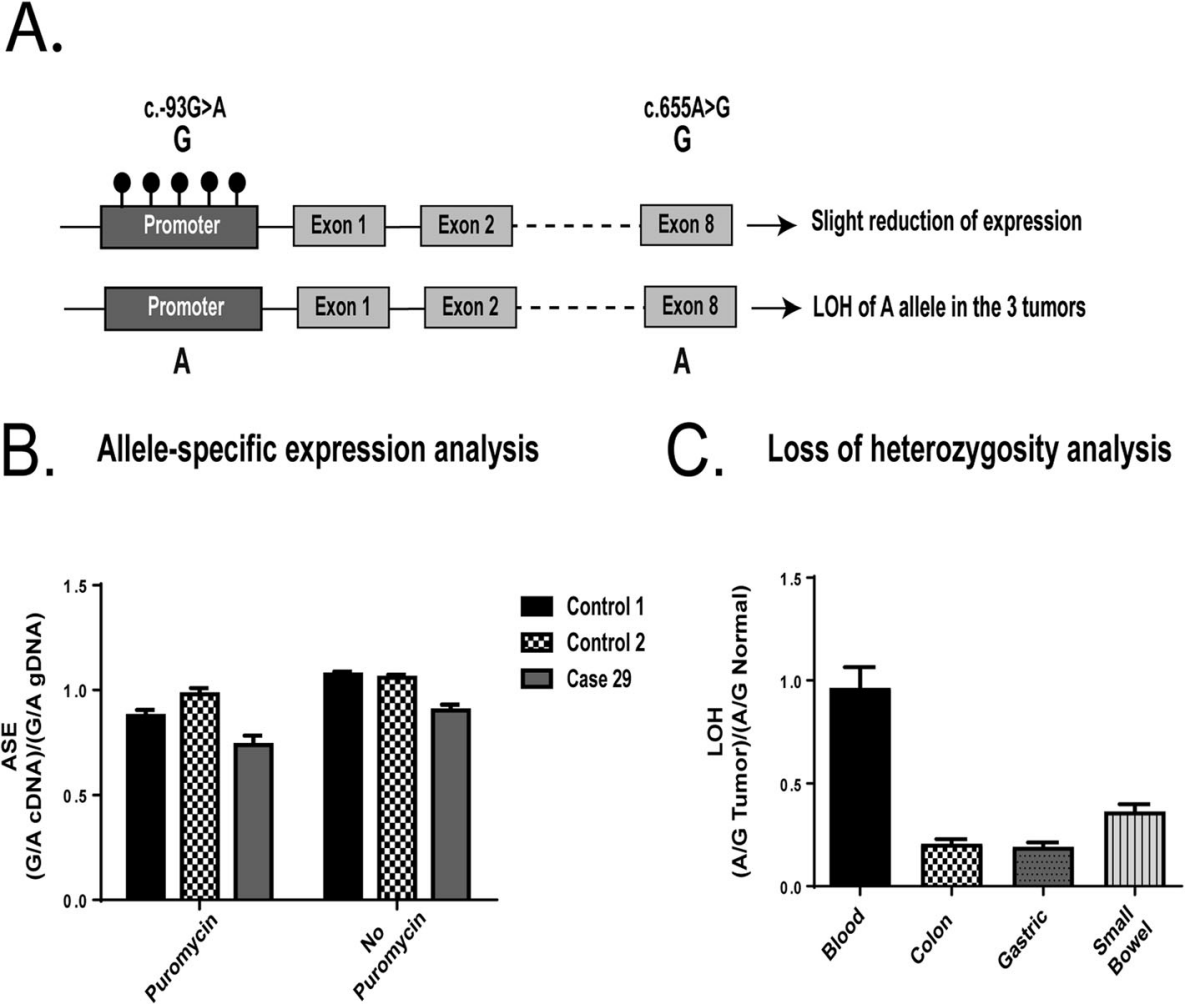
Analysis of the dose of *MLH1* alleles at rs1799977 (c.655A>G). **a** Schematic representation of the *MLH1* epimutation found in case 29. Promoter methylation is associated with the c.-93G allele (in approximately 2% of cells), located in cis with exonic c.655G allele, which showed subtle reduced transcriptional activity in blood. In concordance, the *MLH1* c.655A allele showed LOH in the three tumor lesions developed by the proband. **b** Allele-specific expression (ASE) analysis at *MLH1* c.655G>A in cDNA derived from leukocytes of case 29 and two heterozygous controls. A slightly diminished expression of the c.655G allele was observed. **c** Loss of heterozygosity (LOH) analysis in tumors from case 29. Allele specific values show loss of the c.655A allele in all three tumors

Sequencing of *MLH1* cDNA clones showed that the mosaic methylation of the c.-93G allele was in phase with the c.655G allele in patient 29 (Fig. 3a). Consistent with the low percentage of methylation associated with the c.-93G/c.655G allele, a slight reduction in the expression of this allele was observed in *MLH1* transcripts, as compared to the *MLH1* expression in two control individuals (Fig. 3b). Accordingly, the three tumors showed somatic LOH on the opposite allele (c.655A) (Fig. 3c).

**Fig. 4:**
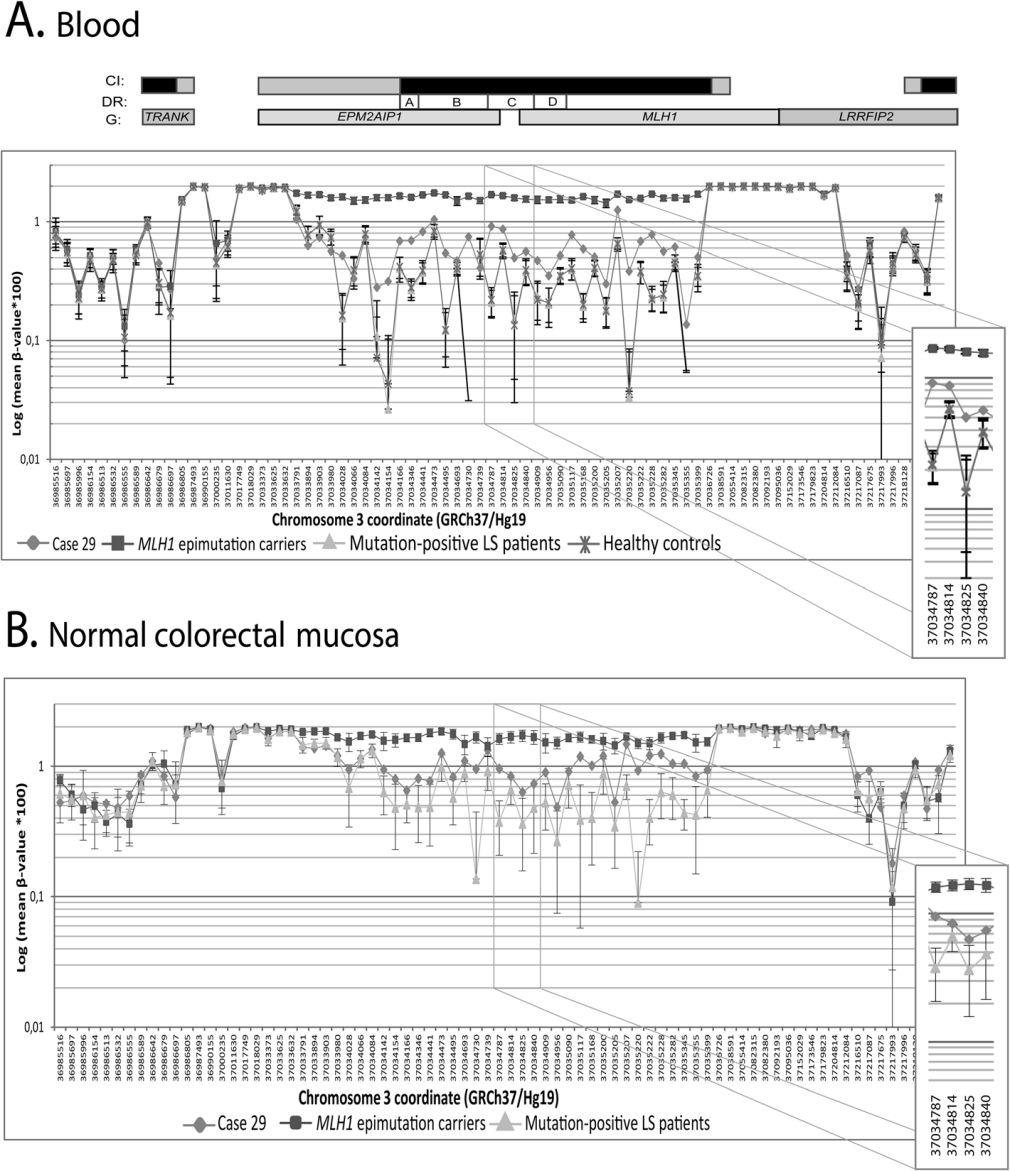
Representation of the differentially methylated region across the *MLH1* locus in blood and colorectal mucosa from case 29, *MLH1* epimutation carriers (*n* = 12), Lynch syndrome genetic mutation carriers (*n* = 61), and healthy controls (*n* = 41). Representation of the differentially methylated region across the *MLH1* locus in blood DNA (**a**) and in normal colorectal mucosa (**b**). β-values obtained from Infinium 450k Human Methylation array analysis are displayed as a log (mean β-value × 100) against the genomic coordinate for each CpG site interrogated. The relative locations of the CpG sites are not drawn to scale. CpG sites are located between Chr3:36,985,516-37,219,077 coordinates. Above, CpG islands (CI) are represented as black rectangles and their shores are represented in gray. The location of the Deng regions (DR) A, B, C and D of the *MLH1* promoter are indicated by white rectangles. Genes (G) containing the displayed CpG sites are represented as gray rectangles, using the Ensembl GRCh37 database as the reference for gene coordinates

Global methylome array analysis revealed slightly higher levels of methylation across the *MLH1* promoter in blood and normal colonic mucosa samples from patient 29 compared to controls (Fig. 4a, b), encompassing the same region previously shown to be differentially methylated in carriers of a constitutional *MLH1* epimutation [9].

Of note, germline pathogenic variants were not found in the coding regions of hereditary cancer genes including *MLH1* (its promoter sequence was also analyzed) in patient 29 (Additional file 8: Table S3). Furthermore, no *MLH1* germline copy number alterations were identified (Additional file 9: Figure S5). These findings, coupled with the lack of a cancer family history, suggest that patient 29 is the carrier of a primary epimutation.

## Discussion

We report the finding of low-level constitutional *MLH1* epigenetic mosaicism in a woman who suffered from three sequential *MLH1*-methylated tumor lesions of the upper abdominal area in her early twenties. A comprehensive analytical approach that combined highly sensitive MS-MCA with clonal bisulfite sequencing and global methylome array analysis confirmed the presence of dense allele-specific methylation spanning the entire *EPM2AIP1-MLH1* CpG island in a low proportion (~ 1%) of the *MLH1* alleles (Additional file 10: Figure S6).

Our MS-MCA approach allowed the robust and highly sensitive detection of *MLH1* methylation in blood, whereas no evidence of methylation was detected in controls. In contrast, pyrosequencing and MS-MLPA, widely used in clinical diagnostics for *MLH1* methylation detection [12, 14, 23, 24], display a lower analytical sensitivity (5–10%) than MS-MCA (1%) [19], potentially overlooking low-level epigenetic mosaicism. Moreover, the background signal observed in pyrosequencing analyses could account for the high proportion (78%) of low-level (< 10%) methylation levels previously reported in healthy controls [17]. Of note, the presence of constitutional methylation was only confirmed in one (case 29) of the five patients showing methylation levels between 1 and 4% by MS-MLPA (Additional file 2: Table S1). Since MS-MLPA is based on methylation-sensitive enzymes, incomplete digestion may account for this apparent inconsistency.

Constitutional epigenetic mosaicism in *MLH1* is often observed (reviewed in [9]), in contrast to MMR genetic mosaicism that has been rarely reported [25–27]. Although several cases with low-level epigenetic mosaicism of *MLH1* (≤ 10% methylation) have been reported [10, 13–16], to date only seven were validated by other techniques. These reported mosaic cases have shown highly variable clinical phenotypes (Additional file 11: Table S4), being the case identified herein the most expressive. Interestingly, our patient was clinically treated considering the three tumor lesions as non-related primary cancers. Retrospectively, a shared origin of the tumors is highly suggested based on the high percentage of shared mutations [28–30] and the same immunohistochemistry CK20-negative pattern, very rare in CRC [31]. This highlights the clinical complexity of the case.

We did not identify any genetic alteration associated with the epimutated allele in case 29, including copy number variations, promoter variants within the C-D promoter regions, or other sequencing variants within *MLH1* by Sanger or next-generation sequencing using mosaicism pipelines. To date, nine families carrying a secondary epimutation have been described (reviewed in [8–10]). In one case, the epimutation was present on a low proportion of alleles (< 10%), associated with the silent variant c.27G>A [10]. Although we cannot completely rule out that genetic alterations have been missed, the lack of family history is also compatible with a de novo primary epimutation. Furthermore, the detection of similar levels of *MLH1* methylation in tissues derived from the three embryonic layers suggests that the epimutation arose either during early embryogenesis or as a germline error that was partially erased during early embryogenesis [5]. 

The phenotypic expressivity of patient 29 contrasts with the subtle functional impact on *MLH1* expression, in accordance with the low methylation levels. The possibility of constitutional MMR deficiency in case 29 was formally discarded because of the absence of germline mutations in the promoter and coding regions of *MLH1* and the conserved biallelic *MLH1* transcription and MLH1 protein expression in normal tissues. Furthermore, no pathogenic alterations in other hereditary cancer genes were detected, although other genetic and/or environmental factors could be playing a role in the observed phenotype.

In contrast to high-level methylation, the potential contribution of low-level mosaic methylation in blood to cancer risk remains to be properly assessed [6]. The combination of soma-wide allelic methylation and associated transcriptional silencing in a small proportion of cells is consistent with the initiation of carcinogenesis from the ~ 2% cells that contained the epimutation. Eventually, the somatic loss of the functional (non-methylated) allele and its clonal expansion would give rise to each of the *MLH1*-methylated tumors.

Based on the clinical phenotype and the molecular profile, intensive surveillance of metachronous gastrointestinal and gynecological tumors has been recommended to patient 29 [32]. Also, predictive epigenetic testing should be proposed to family members. Unless stable inheritance of hypermethylation could be demonstrated in descendants (as previously reported [14]), the estimation of cancer risk in relatives should be cautious in the absence of an established causal mechanism.

Taking into account the present report, we have identified three bona fide *MLH1* epimutation carriers (two previously reported in [18] and one in the present study) among 71 (4.2%) patients with *MLH1*-methylated CRC and in three of 20 (15%) patients with early onset or multiple tumors (Additional file 1: Figure S1). In all, *MLH1* epimutations represent 1% of all LS cases in our series, including case 29 identified by the use of highly sensitive techniques.

## Conclusion

In summary, we have identified a bona fide case of low-level *MLH1* epigenetic mosaicism by using highly sensitive *MLH1* methylation analysis. Considering the obtained results, we strongly recommend the use of highly sensitive techniques for screening of constitutional methylation in patients diagnosed with early onset and/or multiple *MLH1*-methylated tumors. The eventual identification and characterization of additional cases will be critical to ascertain the cancer risks associated with epigenetic mosaicism.

## Supplementary information

**Additional file 1.** **Figure S1.** Schematic representation of the origin of the 18 cases included in this study. CRC: colorectal cancer.

**Additional file 2: Table S1.** Clinical and molecular features of the patients with *MLH1* methylated tumors included in this analysis.

**Additional file 3.** Supplementary Methods.

**Additional file 4: Figure S2.** *MLH1* promoter methylation analysis by Methylation-Specific Melting Curve Analysis (MS-MCA). **A)** Analytical sensitivity of the promoter C region. The assay displays a sensitivity threshold of 1%. **B)** Analytical sensitivity of the promoter D region. The assay shows sensitivity around 10%. **C)** Methylation analysis in blood from 10 healthy controls for the *MLH1* C-region. All of them show the same melting curve pattern as the unmethylated control sample, indicating absence of methylation in healthy controls. **D)** Methylation analysis in blood from 18 patients harboring *MLH1* methylated tumors for the promoter C region. Only case 29 displays low levels of methylation (around 1%). **E)** Methylation analysis in tumor and normal gastrointestinal tissues of case 29. **F)** Methylation analysis in buccal mucosa of case 29. **G)** Methylation analysis in skin fibroblasts of case 29.

**Additional file 5: Figure S3.** *MLH1* methylation analysis of the promoter C-region and intron 1 by pyrosequencing. **A)** and **B)** Analytical sensitivity analysis for the detection of methylation in *MLH1* C-region (A) and intron 1 (B). The detection limits for both regions are 4% and 5% respectively, enabling the detection of positive samples as those with methylation values greater than 4 or 5%. **C)** and **D)** Methylation analysis in blood from case 29 and healthy controls (*n*=10 - 20) for the *MLH*1 C-region (C) and intron 1 (D). **E) and F)** Methylation analysis in normal colorectal mucosa from case 29 and Lynch patients (*n*=4) for *MLH1* C-region (E) and intron 1 (F). **G)** and **H)** Methylation analysis in normal small bowel mucosa and gastric mucosa from case 29 for *MLH1* C-region (G) and intron 1 (H). **I) and J)** Methylation analysis in normal and gastrointestinal tumor tissues in case 29 for *MLH1* C-region (I) and intron 1 (J).

**Additional file 6:** **Figure S4.** Immunohistochemical characterization of gastrointestinal tumor lesions from patient 29. All tumor lesions are well-differentiated adenocarcinomas with a variable but not predominant mucinous component. All of them present loss of expression of the cytokeratin markers CK7 and CK20. The transcription factor CDX2 shows strong and diffused positive staining. The expression of the membrane-bound proteins MUC1 and MUC5 is also positive in all tumors but with diffused and lower intensity staining, whereas the MUC2 shows intense, focal and heterogeneous expression. According to this characterization, the three tumors show the same immunohistochemical staining pattern. Objective magnification is 20X for all images. HE, hematoxylin-eosin.

**Additional file 7: Table S2.** *MLH1* methylation assessed by MS-MLPA in samples from case 29.

**Additional file 8:** **Table S3.** Variants identified in the mutational analysis of hereditary cancer genes in case 29.

**Additional file 9: Figure S5.** Analysis of structural aberrations in case 29. **A)** Genome-wide SNP array profiling of blood DNA from case 29 is shown as Circos plots. Circos plot was divided into three concentric circles. Chromosomes are represented at the external circle with their centromeres painted in red. In the middle circle, external allelic peaks mark homozygous SNPs and internal allelic peaks heterozygous ones. Internal circle tracks log2 copy number lane: middle points indicate diploid genomic material; upper points, gains of genomic material and lower points, losses. Patient 29 displayed a diploid pattern throughout her genome without signs of loss-of-heterozygosity. **B)** CNV analysis in the *MLH1* region of patient 29 by custom CGH array. Genes located in the analyzed region are represented at the bottom of the figure. Probes are displayed as green dots in a log2 graph. Gains and losses of genetic material are considered when more than five consecutive probes reach values of 2 or − 2, respectively. No CNV abnormalities were identified.

**Additional file 10:** **Figure S6.** Schematic representation of the methodological strategy and summary of the obtained results.

**Additional file 11: Table S4.** Reported patients with *MLH1* epigenetic mosaicism at low proportion **(≤10%).** (*) According to the obtained results the three tumor lesions were clonally related.

**Additional file 12: Table S5.** A. Primers and conditions. B. Localization of the probes and regions analyzed in the study of *MLH1* methylation. The *EMP2AIP1-MLH1* CpG island (colored in dark purple) encompass the *MLH1* promoter and intron 1.

## Data Availability

The data discussed in this publication is accessible through GEO Series accession number GSE131541 and GSE107353 (https://www.ncbi.nlm.nih.gov/geo/query/acc.cgi?acc=GSE131541 and https://www.ncbi.nlm.nih.gov/geo/query/acc.cgi?acc=GSE107353).

## Abbreviations

CRC: Colorectal cancer
LOH: Loss of heterozygosity
LS: Lynch syndrome
MMR: Mismatch repair
MS-MCA: Methylation-specific melting curve analysis
MS-MLPA: Methylation-specific multiplex ligation-dependent probe amplification
PBL: Peripheral blood leukocytes
SNuPE: Single-nucleotide primer extension
WGA: Whole Genome Amplification
