# Supplementary material for: Highly sensitive MLH1 methylation analysis in blood identifies a cancer patient with low-level mosaic MLH1 epimutation
Source: Clin Epigenetics. 2019 Nov 28;11:171. doi: 10.1186/s13148-019-0762-6 (PMC6883525; doi:10.1186/s13148-019-0762-6)
Supplement: Supplementary file 1 — Additional file 1. Figure S1. Schematic representation of the origin of the 18 cases included in this study. CRC: colorectal cancer. [file 13148_2019_762_MOESM1_ESM.pdf]

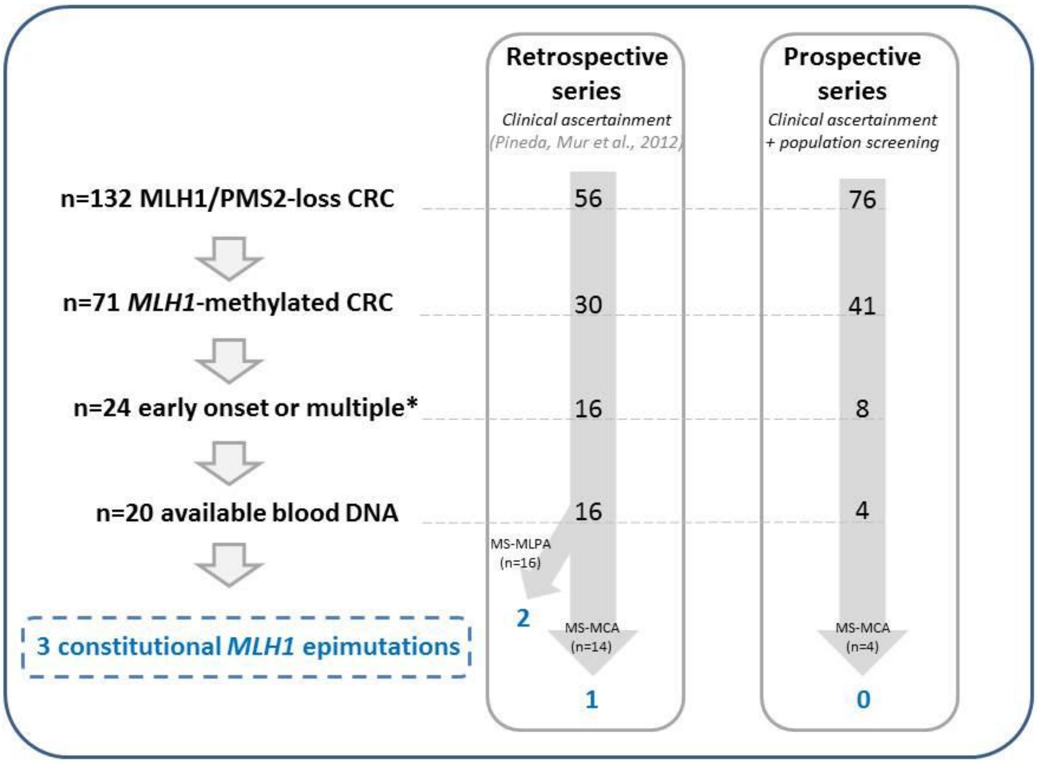

Figure S1. Schematic representation of the origin of the 18 cases included in this study. CRC: colorectal cancer.
