# Supplementary material for: Highly sensitive MLH1 methylation analysis in blood identifies a cancer patient with low-level mosaic MLH1 epimutation
Source: Clin Epigenetics. 2019 Nov 28;11:171. doi: 10.1186/s13148-019-0762-6 (PMC6883525; doi:10.1186/s13148-019-0762-6)
Supplement: Supplementary file 2 — Additional file 2: Table S1. Clinical and molecular features of the patients with MLH1 methylated tumors included in this analysis. [file 13148_2019_762_MOESM2_ESM.pdf]

Table 1. Clinical and molecular features of the patients with *MLH1* methylated tumors included in this analysis

ID patient	Gender	Clinical Criteria	Blood: *MLH*1 methylation assessed by MS-MLPA (%)			Tumor age of onset	Tumor: *MLH1* methylation assessed by MS-MLPA (%)			Other tumors
			C region	D region	Intron 1		C region	D region	Intron 1	
2[R]	F	BC	0	0	0	CRC (49)	24,9	36,9	40,3	
3[R]	M	BC	0	0	0	CRC (37)	29,3	31,7	106	
5[R]	M	BC	0	0	0	CRC (50)	28,6	33,6	49	TC (49)
7[R]	M	BC	0	0	0	CRC (42)	24,1	25,2	29,3	CRC[met](synch)
8[R]	M	BC	0	0	0	CRC (29)	25,1	27,6	28,5	
9[R]	F	BC	0	0	0	CRC (47)	38,5	34,9	39,7	
16[R]	F	BC	2	0	0	CRC (24)	57,5	75,1	53,6	
18[R]	M	BC	0	0	0	CRC (48)	32,8	34,8	43,7	
21[R]	F	BC	0	0	0	CRC (58)	40,6	66,6	74,3	3 CRC (synch)
23[R]	F	BC	0	0	0	CRC (47)	20,3	39,3	39,9	
24[R]	F	BC	0	0	0	CRC (59)	11,4	20,6	37,4	CRC (29)
27[R]	M	BC	0	0	0	CRC (47)	40,1	21,6	64,0	
28[R]	M	BC	2	0	0	CRC (31)	32	33	35,6	
29[R]	F	BC	3,6	2,9	3	CRC (22)	76,9	101,6	66,0	SB[met](24), GC[met](25)
30[P]	F	BC	0	0	0	CRC (39)	71	67	76,0	
31[P]	F	BC	0	0	0	CRC (47)	43	39	54,4	
32[P]	F	BC	3	0	0	CRC (47)	34	23	37,2	
33[P]	F	BC	2,4	0	0	CRC (63)	49	41	55,1	CRC (41)

Abbreviations: BC, Bethesda Criteria; M, male; F, female; CRC, colorectal cancer; TC, testicular cancer; SB, small bowel cancer; GC, gastric cancer; synch, synchronous; R: retrospective series, patient ID from the original publication Pineda *et al.,* 2012; P: prospectively collected series.
