# Supplementary material for: Highly sensitive MLH1 methylation analysis in blood identifies a cancer patient with low-level mosaic MLH1 epimutation
Source: Clin Epigenetics. 2019 Nov 28;11:171. doi: 10.1186/s13148-019-0762-6 (PMC6883525; doi:10.1186/s13148-019-0762-6)
Supplement: Supplementary file 3 — Additional file 3. Supplementary Methods. [file 13148_2019_762_MOESM3_ESM.docx]

**SUPPLEMENTARY METHODS**

**Patients and sample processing**

From a series of 132 colorectal cancer (CRC) cases harboring immunohistochemical loss of expression of MLH1 and PMS2 in their tumor, of which 71 were *MLH1*-methylated, 18 cases who presented *MLH1*-methylated CRC below 50 years of age, or multiple tumors below 60 years of age, were included in this study (**Additional file 1: Figure S1; Additional file 2: Table S1**). Fourteen of the 18 patients were previously reported [1] and the remaining four cases were subsequently identified **Additional file 1: Figure S1; Additional file 2: Table S1**). Clinico-pathological data was collected, including age at cancer diagnosis, tumor location, and *MLH1* methylation status in tumor and blood DNA (**Additional file 2: Table S1**). All 18 cases fulfilled Bethesda criteria; four were diagnosed with CRC below 35 years of age, and six presented with multiple tumors. Cases 7 and 21 developed synchronous tumors. Cases 7 and 29 were positive for *MLH1* methylation in more than one of their tumors (**Additional file 2: Table S1**).

Samples of peripheral blood leukocyte (PBL) DNA were obtained from the biobank at the Catalan Institute of Oncology (ICO). For each formalin-fixed paraffin embedded (FFPE) tumor tissue specimen, 10-20 x 10-μm sections were cut from a single block and macrodissected with a scalpel where needed to enrich for tumor cells. After deparaffinization using Deparaffinization Solution (Qiagen, Hilden, Germany), DNA was isolated using the QIAmp DNA FFPE Tissue Kit (Qiagen) according to the manufacturer's instructions. Skin fibroblasts were cultured as reported [1] and DNA extraction was carried out using the Wizard Genomic DNA purification Kit. Buccal mucosa was obtained using Isohelix DNA buccal swabs and DNA was isolated by phenol/chloroform extraction.

**Immunohistochemical staining analysis**

Formalin-fixed, paraffin-embedded tissue sections representative of the tumors were studied using standard immunohistochemistry techniques. The mouse primary antibodies used were the following: anti-CK7 (ROCHE VENTANA, (SP52) Rabbit monoclonal primary antibody), anti-CK20 (ROCHE VENTANA,(SP33) Rabbit monoclonal primary antibody), anti-CDX2 (ROCHE VENTANA, (EPR2764Y) Rabbit monoclonal primary antibody), anti-MUC1 (ROCHE VENTANA, (H23) Mouse monoclonal primary antibody), anti-MUC2 (ROCHE VENTANA, (MRQ-18) Mouse monoclonal primary antibody) and anti-MUC5 (ROCHE VENTANA, (MRQ-19) Mouse monoclonal primary antibody).

**Somatic and germline methylation testing**

***Methylation-speciﬁc multiplex ligation-dependent probe ampliﬁcation (MS-MLPA)***

The methylation status at the *MLH1* promoter was assessed using the SALSA MLPA ME011 Mismatch Repair genes probemix (MRC-Holland), according to the manufacturer’s instructions. The kit SALSA MLPA ME011 Mismatch Repair genes probemix (MRC-Holland) includes ﬁve probe pairs within the *MLH1* CpG island (with the respective HhaI sites located at -659, -383, -246, -13 and +208 relative to the start codon; GenBank accession number U26559) that cover ﬁve independent regions: regions A to D of the promoter and intron 1 (**Additional file 12: Table S5**). DNA from the RKO CRC cell line was used as the *MLH1* methylation-positive control. The amplification products were run on an ABI Prism 3130 DNA sequencer and analyzed using GeneMapper v4.0 (Applied Biosystems).

**Methylation-specific melting curve analysis (MS-MCA)**

One µg of DNA was treated with sodium bisulfite using the EZ DNA Methylation-Gold Kit (Zymo Research, Orange, CA, USA) under the manufacturer’s conditions. One μl of bisulfite treated DNA was used to amplify Deng’s C and D regions in a nested PCR reaction using MegaMix double solution (Microzone Ltd., UK). Each promoter region was preamplified using external primers (**Additional file 12: Table S5**). The nested PCR was carried out in a LightCycler 480 II (Roche) using 1µl of amplified *MLH1* promoter fragment, Light Cycler 480 SYBR Green I Master Kit (Roche) and internal primers (**Additional file 12: Table S5**). Results were analyzed with the Light Cycler ®480 software version 1.5.1 [1].

Since MS-MLPA had shown an analytical sensitivity of 5-10% for *MLH1* methylation detection [2] in the C/D promoter regions (previously associated with *MLH1* transcriptional silencing [3], MS-MCA was optimized for the highly sensitive analysis of methylation. The analytical sensitivity of MS-MCA for the detection of *MLH1* promoter methylation was assessed using serial dilutions (100, 75, 50, 25, 10, 5, 4, 3, 2 and 1%) of the RKO cell line (biallelic *MLH1* methylation; 100% methylated) into unmethylated (0% methylated) WGA (Whole Genome Amplification obtained by using Illustra GenomiPhi HY DNA Amplification Kit, GE Healthcare Life Sciences) DNA. WGA DNA and RKO DNA showed melting peaks at 73ºC and 77ºC, respectively. Methylation levels in DNA samples from patients and controls were evaluated alongside the reconstitutions of RKO in WGA within the same experiment. The MS-MCA technique demonstrated analytical sensitivity to detect methylation of 1% in the C region and 10% in the D region (**Additional file 4: Figure S2A-B**).

***Pyrosequencing***

One μl of bisulfite-converted DNA was used in a PCR reaction for the amplification of regions C and intron 1 of *MLH1* using Immolase^TM^ DNA polymerase kit (Bioline, UK) and MegaMix double solution (Microzone Ltd., UK), respectively, and biotin-labeled primers (designed with MethPrimer; **Additional file 12: Table S5**). Purification and subsequent processing of the biotinylated single-stranded DNA was performed according to the manufacturer's recommendations at the PyroMark Q24 Vacuum PrepWorkstation (Qiagen). Pyrosequencing reaction was performed using each specific sequencing primer (**Additional file 12: Table S5**) on a PyroMark Q24 pyrosequencer system using the Pyromark Gold Q26 Reagents kit. The sequences analyzed were GAGYGGATAGYGATTTTTAAYGYGTAAGYGTA for the promoter C region and YGATTTAAYGGGTYGYGTTATTTAATGGYGYGGATAYGT for intron 1. The methylation in targeted CpG sites was analyzed using the software PyroMark Q24 2.0.6. Each sample was run in triplicate. Methylation at each specific CpG was calculated as the mean of all triplicates and statistical analysis was performed using Graphpad Prism. The limit of detection of *MLH1* methylation was assessed using serial dilutions of the RKO cell into unmethylated WGA DNA as described above, and calculated as the mean methylation signal of unmethylated DNA (S) plus three times its standard deviation (S+3*SD). The technique demonstrated a sensitivity of 4% in the C region and 5% in intron 1 (**Additional file 5: Figure S3A-B**).

***Clonal sequencing***

Clonal bisulfite sequencing of a fragment of the *MLH1* promoter was used to determine the allelic methylation profile in bisulfite modified PBL DNA from *MLH1* epimutation carriers. Primers unbiased with respect to methylation status were used to amplify the promoter region encompassing variant c.-93G>A (**Additional file 12: Table S5**) and PCR products were cloned in *E. coli* cells using the pGEM-T easy vector system (Promega) to separate the amplicons into individual alleles [4]. The inserts from individual colonies were sequenced using vector primers. The methylation status at each individual CpG site was determined using SeqMan (DNASTAR). The number of clones needed to acquire statistical power was calculated using the formula n=[*z*^2^ x *p* x (1-*p*)]/*e*^2^, where *z* is the mean value deviation, *p* is the expected proportion of methylated clones and *e* is the error range. Considering *p* as 0.03 (based on previous results), *e* as 0.02 and z as 1.96, the analysis of at least 280 clones was performed in order to achieve a confidence interval between 0,01–0,05.

A 1039bp cDNA fragment (from the 5’UTR to exon 8) was amplified using TaKaRa LA Taq® DNA Polymerase kit (Takara) (primers detailed in **Additional file 12: Table S5**). Clonal sequencing of the *MLH1* cDNA fragment was used to determine the phase between variants c.-93G>A and c.655A>G. PCR products were cloned into *E. coli* cells using pGEM-T easy vector system (Promega). Individual clones were sequenced using vector and internal primers (**Additional file 12: Table S5**).

***Genome wide methylation analysis***

Genome wide methylation profiling was performed using the Infinium Human Methylation 450K Beadchip (Illumina) as previously described [4]. One microgram of bisulfite treated DNA from blood and normal colorectal mucosa was hybridized in addition to in vitro methylated and unmethylated DNAs as internal quality control samples. Sample scanning was performed using the HiScan platform (Illumina), which has a laser scanner with two colours (532nm/660nm). The relative intensity of each dye was analyzed using the GenomeStudio software (Methylation Module). For each analyzed CpG site, a β-value was obtained depending on the florescence intensity. β values took measures between 0 (unmethylated) and 1 (fully methylated). Results were compared with data previously obtained of the same platform from 41 healthy controls, 51 mutation-positive Lynch syndrome patients and 12 constitutional *MLH1* epimutation carriers [4].

**Mutational analysis of hereditary cancer genes including *MLH1***

Hereditary cancer genes (including *MLH1*) were screened for rare and mosaic variants. Mutational analysis was performed using two customized NGS panels: a panel of 26 CRC-associated genes [5] and a panel of 126 hereditary cancer genes (I2HCP v2.1) [6]. Identified variants were filtered against common single-nucleotide polymorphisms (MAF>1 according to GnomAD, ExAC and ESP databases). The presence of mosaic *MLH1* genetic variants, that is the presence of *MLH1* genetic variants at a lower than 50% proportion of alleles, was further explored by applying mosaicism detection pipelines (alternative allele ratio ≥0.01).

The presence of structural alterations in *MLH1* was tested for using the SALSA MLPA P003-B1 MLH1/MSH2 Mismatch Repair genes probemix (MRC-Holland), by a custom-designed high definition CGH array [4] and by a custom SNP array [7].

**Statistical analysis**

The results obtained were analyzed using the non-parametric Mann–Whitney U test for continuous data. All reported P values are 2 sided, and P<0.05 was considered significant.

**References:**

1. Pineda M, Mur P, Iniesta MD, Borràs E, Campos O, Vargas G, et al. MLH1 methylation screening is effective in identifying epimutation carriers. Eur J Hum Genet. 2012;20(12):1256-64.

2. Gausachs M, Mur P, Corral J, Pineda M, González S, Benito L, et al. MLH1 promoter hypermethylation in the analytical algorithm of Lynch syndrome: a cost-effectiveness study. Eur J Hum Genet. 2012;20(7):762-8.

3. Deng G, Chen A, Hong J, Chae HS, Kim YS. Methylation of CpG in a small region of the hMLH1 promoter invariably correlates with the absence of gene expression. Cancer Res. 1999;59(9):2029-33.

4. Dámaso E, Castillejo A, Arias MDM, Canet-Hermida J, Navarro M, Del Valle J, et al. Primary constitutional MLH1 epimutations: a focal epigenetic event. Br J Cancer. 2018;119(8):978-87.

5. Vargas-Parra GM, González-Acosta M, Thompson BA, Gómez C, Fernández A, Dámaso E, et al. Elucidating the molecular basis of MSH2-deficient tumors by combined germline and somatic analysis. Int J Cancer. 2017;141(7):1365-80.

6. Castellanos E, Gel B, Rosas I, Tornero E, Santín S, Pluvinet R, et al. A comprehensive custom panel design for routine hereditary cancer testing: preserving control, improving diagnostics and revealing a complex variation landscape. Sci Rep. 2017;7:39348.

7. Castellsagué J, Gel B, Fernández-Rodríguez J, Llatjós R, Blanco I, Benavente Y, et al. Comprehensive establishment and characterization of orthoxenograft mouse models of malignant peripheral nerve sheath tumors for personalized medicine. EMBO Mol Med. 2015;7(5):608-27.
