# Supplementary material for: Highly sensitive MLH1 methylation analysis in blood identifies a cancer patient with low-level mosaic MLH1 epimutation
Source: Clin Epigenetics. 2019 Nov 28;11:171. doi: 10.1186/s13148-019-0762-6 (PMC6883525; doi:10.1186/s13148-019-0762-6)
Supplement: Supplementary file 4 — Additional file 4: Figure S2. MLH1 promoter methylation analysis by Methylation-Specific Melting Curve Analysis (MS-MCA). A) Analytical sensitivity of the promoter C region. The assay displays a sensitivity threshold of 1%. B) Analytical sensitivity of the promoter D region. The assay shows sensitivity around 10%. C) Methylation analysis in blood from 10 healthy controls for the MLH1 C-region. All of them show the same melting curve pattern as the unmethylated control sample, indicating absence of methylation in healthy controls. D) Methylation analysis in blood from 18 patients harboring MLH1 methylated tumors for the promoter C region. Only case 29 displays low levels of methylation (around 1%). E) Methylation analysis in tumor and normal gastrointestinal tissues of case 29. F) Methylation analysis in buccal mucosa of case 29. G) Methylation analysis in skin fibroblasts of case 29. [file 13148_2019_762_MOESM4_ESM.pdf]

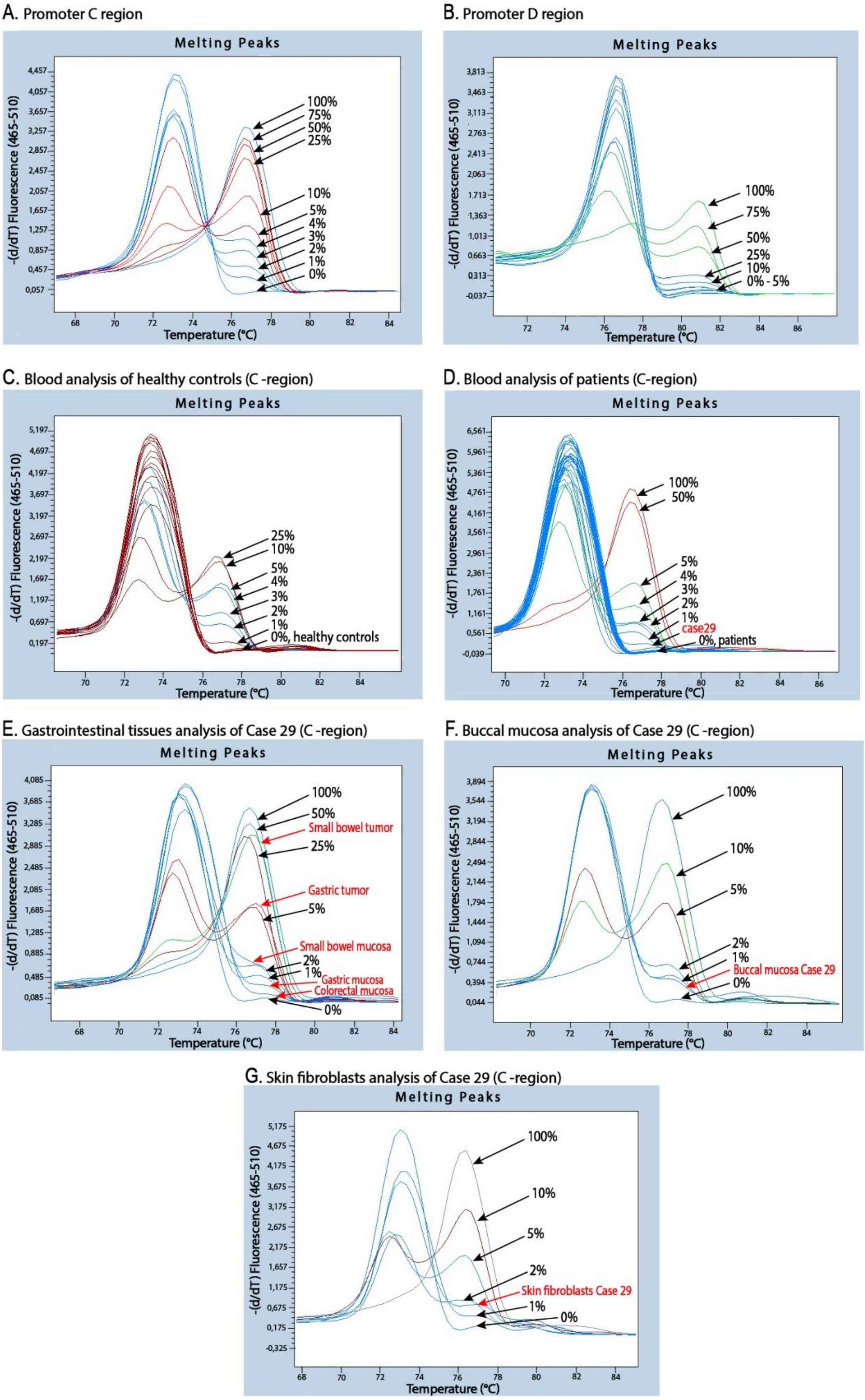
A. Promoter C region
Melting Peaks
100%
75%
50%
25%
10%
5%
4%
3%
2%
1%
0%
-(d/dT) Fluorescence (465-510)
Temperature (°C)
B. Promoter D region
Melting Peaks
100%
75%
50%
25%
10%
0% - 5%
-(d/dT) Fluorescence (465-510)
Temperature (°C)
C. Blood analysis of healthy controls (C -region)
Melting Peaks
25%
10%
5%
4%
3%
2%
1%
0%, healthy controls
-(d/dT) Fluorescence (465-510)
Temperature (°C)
D. Blood analysis of patients (C-region)
Melting Peaks
100%
50%
5%
4%
3%
2%
1%
case29
0%, patients
-(d/dT) Fluorescence (465-510)
Temperature (°C)
E. Gastrointestinal tissues analysis of Case 29 (C -region)
Melting Peaks
100%
50%
Small bowel tumor
25%
Gastric tumor
5%
Small bowel mucosa
2%
1%
Gastric mucosa
Colorectal mucosa
0%
-(d/dT) Fluorescence (465-510)
Temperature (°C)
F. Buccal mucosa analysis of Case 29 (C -region)
Melting Peaks
100%
10%
5%
2%
1%
Buccal mucosa Case 29
0%
-(d/dT) Fluorescence (465-510)
Temperature (°C)
G. Skin fibroblasts analysis of Case 29 (C -region)
Melting Peaks
100%
10%
5%
2%
Skin fibroblasts Case 29
1%
0%
-(d/dT) Fluorescence (465-510)
Temperature (°C)

Figure S2. *MLH1* promoter methylation analysis by Methylation-Specific Melting Curve Analysis (MS-MCA). A) Analytical sensitivity of the promoter C region. The assay displays a sensitivity threshold of 1%. **B)** Analytical sensitivity of the promoter D region. The assay shows sensitivity around 10%. **C)** Methylation analysis in blood from 10 healthy controls for the *MLH1* C-region. All of them show the same melting curve pattern as the unmethylated control sample, indicating absence of methylation in healthy controls. **D)** Methylation analysis in blood from 18 patients harboring *MLH1* methylated tumors for the promoter C region. Only case 29 displays low levels of methylation (around 1%). **E)** Methylation analysis in tumor and normal gastrointestinal tissues of case 29. **F)** Methylation analysis in buccal mucosa of case 29. **G)** Methylation analysis in skin fibroblasts of case 29.
