# Supplementary material for: Highly sensitive MLH1 methylation analysis in blood identifies a cancer patient with low-level mosaic MLH1 epimutation
Source: Clin Epigenetics. 2019 Nov 28;11:171. doi: 10.1186/s13148-019-0762-6 (PMC6883525; doi:10.1186/s13148-019-0762-6)
Supplement: Supplementary file 5 — Additional file 5: Figure S3. MLH1 methylation analysis of the promoter C-region and intron 1 by pyrosequencing. A) and B) Analytical sensitivity analysis for the detection of methylation in MLH1 C-region (A) and intron 1 (B). The detection limits for both regions are 4% and 5% respectively, enabling the detection of positive samples as those with methylation values greater than 4 or 5%. C) and D) Methylation analysis in blood from case 29 and healthy controls (n=10 - 20) for the MLH1 C-region (C) and intron 1 (D). E) and F) Methylation analysis in normal colorectal mucosa from case 29 and Lynch patients (n=4) for MLH1 C-region (E) and intron 1 (F). G) and H) Methylation analysis in normal small bowel mucosa and gastric mucosa from case 29 for MLH1 C-region (G) and intron 1 (H). I) and J) Methylation analysis in normal and gastrointestinal tumor tissues in case 29 for MLH1 C-region (I) and intron 1 (J). [file 13148_2019_762_MOESM5_ESM.pdf]

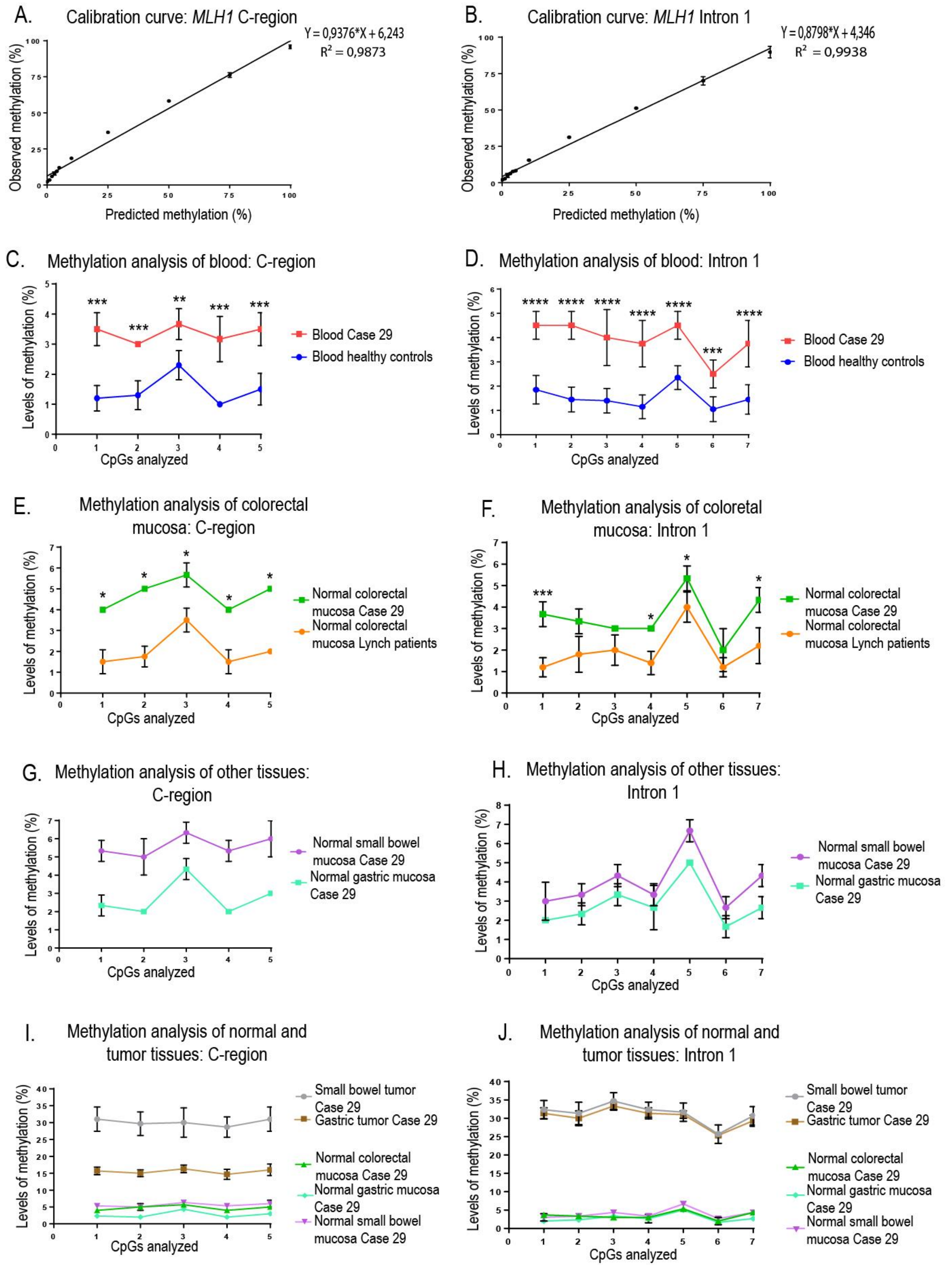

A.
Calibration curve: MLH1 C-region
Y = 0,9376*X + 6,243
R² = 0,9873
Observed methylation (%)
Predicted methylation (%)
B.
Calibration curve: MLH1 Intron 1
Y = 0,8798*X + 4,346
R² = 0,9938
Observed methylation (%)
Predicted methylation (%)
C.
Methylation analysis of blood: C-region
Levels of methylation (%)
CpGs analyzed
Blood Case 29
Blood healthy controls
D.
Methylation analysis of blood: Intron 1
Levels of methylation (%)
CpGs analyzed
Blood Case 29
Blood healthy controls
E.
Methylation analysis of colorectal mucosa: C-region
Levels of methylation (%)
CpGs analyzed
Normal colorectal mucosa Case 29
Normal colorectal mucosa Lynch patients
F.
Methylation analysis of coloretal mucosa: Intron 1
Levels of methylation (%)
CpGs analyzed
Normal colorectal mucosa Case 29
Normal colorectal mucosa Lynch patients
G.
Methylation analysis of other tissues: C-region
Levels of methylation (%)
CpGs analyzed
Normal small bowel mucosa Case 29
Normal gastric mucosa Case 29
H.
Methylation analysis of other tissues: Intron 1
Levels of methylation (%)
CpGs analyzed
Normal small bowel mucosa Case 29
Normal gastric mucosa Case 29
I.
Methylation analysis of normal and tumor tissues: C-region
Levels of methylation (%)
CpGs analyzed
Small bowel tumor Case 29
Gastric tumor Case 29
Normal colorectal mucosa Case 29
Normal gastric mucosa Case 29
Normal small bowel mucosa Case 29
J.
Methylation analysis of normal and tumor tissues: Intron 1
Levels of methylation (%)
CpGs analyzed
Small bowel tumor Case 29
Gastric tumor Case 29
Normal colorectal mucosa Case 29
Normal gastric mucosa Case 29
Normal small bowel mucosa Case 29

Figure S3. ***MLH1*** **methylation analysis of the promoter C-region and intron 1 by pyrosequencing. A) and B)** Analytical sensitivity analysis for the detection of methylation in *MLH1* C-region (A) and intron 1 (B). The detection limits for both regions are 4% and 5% respectively, enabling the detection of positive samples as those with methylation values greater than 4 or 5%. **C) and D)** Methylation analysis in blood from case 29 and healthy controls (n=10 - 20) for the *MLH*1 C-region (C) and intron 1 (D). **E) and F)** Methylation analysis in normal colorectal mucosa from case 29 and Lynch patients (n=4) for *MLH1* C-region (E) and intron 1 (F). **G) and H)** Methylation analysis in normal small bowel mucosa and gastric mucosa from case 29 for *MLH1* C-region (G) and intron 1 (H). **I) and J)** Methylation analysis in normal and gastrointestinal tumor tissues in case 29 for *MLH1* C-region (I) and intron 1 (J).
