# Supplementary material for: Highly sensitive MLH1 methylation analysis in blood identifies a cancer patient with low-level mosaic MLH1 epimutation
Source: Clin Epigenetics. 2019 Nov 28;11:171. doi: 10.1186/s13148-019-0762-6 (PMC6883525; doi:10.1186/s13148-019-0762-6)
Supplement: Supplementary file 6 — Additional file 6: Figure S4. Immunohistochemical characterization of gastrointestinal tumor lesions from patient 29. All tumor lesions are well-differentiated adenocarcinomas with a variable but not predominant mucinous component. All of them present loss of expression of the cytokeratin markers CK7 and CK20. The transcription factor CDX2 shows strong and diffused positive staining. The expression of the membrane-bound proteins MUC1 and MUC5 is also positive in all tumors but with diffused and lower intensity staining, whereas the MUC2 shows intense, focal and heterogeneous expression. According to this characterization, the three tumors show the same immunohistochemical staining pattern. Objective magnification is 20X for all images. HE, hematoxylin-eosin. [file 13148_2019_762_MOESM6_ESM.pdf]

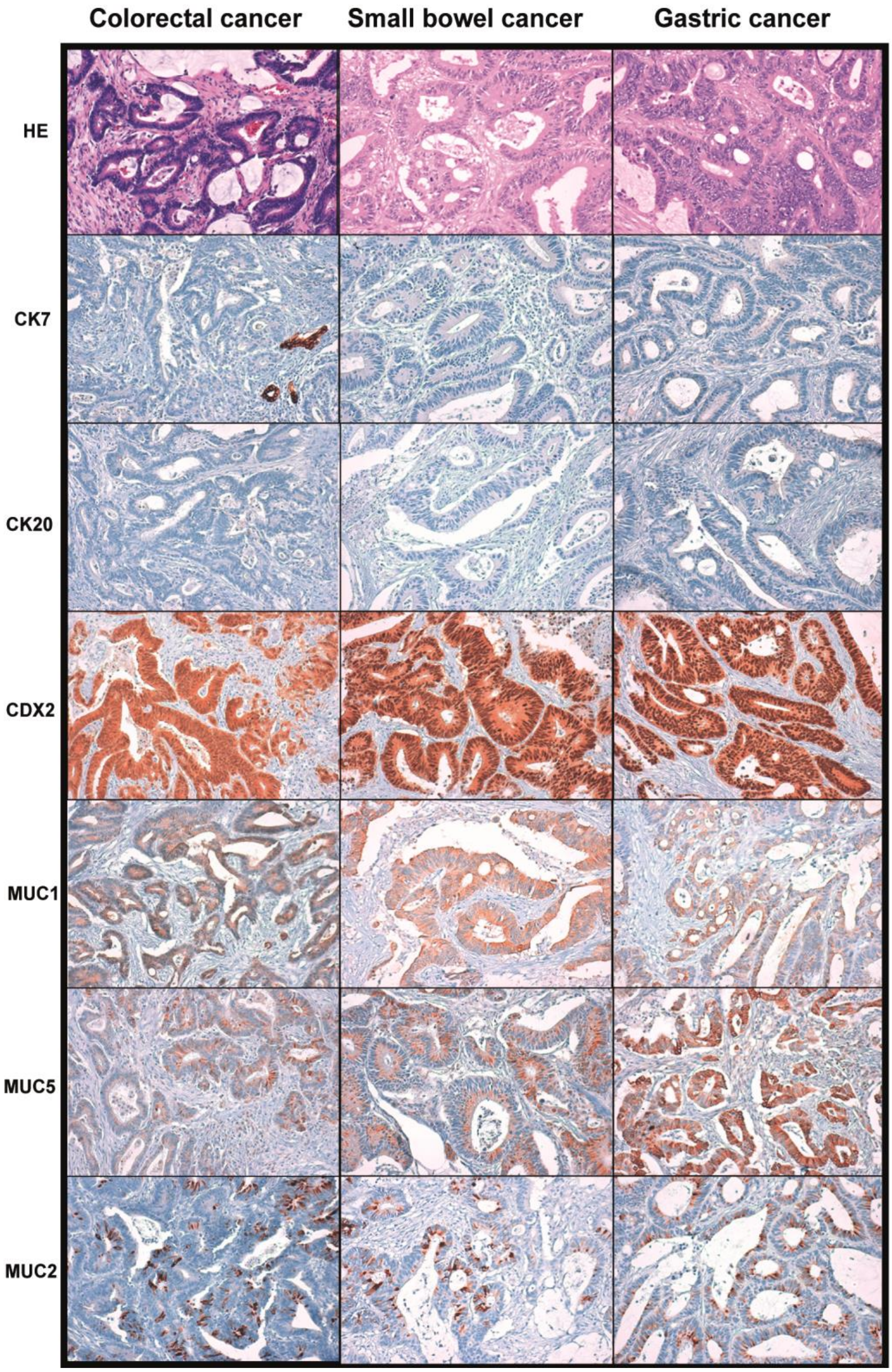

Figure S4. Immunohistochemical characterization of gastrointestinal tumor lesions from patient 29. All tumor lesions are well-differentiated adenocarcinomas with a variable but not predominant mucinous component. All of them present loss of expression of the cytokeratin markers CK7 and CK20. The transcription factor CDX2 shows strong and diffused positive staining. The expression of the membrane-bound proteins MUC1 and MUC5 is also positive in all tumors but with diffused and lower intensity staining, whereas the MUC2 shows intense,

focal and heterogeneous expression. According to this characterization, the three tumors show the same immunohistochemical staining pattern. Objective magnification is 20X for all images. HE, hematoxylin-eosin.
