# Supplementary material for: Highly sensitive MLH1 methylation analysis in blood identifies a cancer patient with low-level mosaic MLH1 epimutation
Source: Clin Epigenetics. 2019 Nov 28;11:171. doi: 10.1186/s13148-019-0762-6 (PMC6883525; doi:10.1186/s13148-019-0762-6)
Supplement: Supplementary file 7 — Additional file 7: Table S2. MLH1 methylation assessed by MS-MLPA in samples from case 29. [file 13148_2019_762_MOESM7_ESM.pdf]

Table S2. ***MLH1*** **methylation assessed by MS-MLPA in samples from case 29**

Sample	Analyzed region (% of methylation)				
	A	B	C	D	Intron 1
Lymphocytes	6	5	3	3	3
Colorectal mucosa	19	6	6	10	14
Colorectal tumor	60	109	80	50	66
Small bowel mucosa	18	3	2	5	14
Small bowel tumor	45	73	33	37	44
Gastric mucosa	14	2	0	3	10
Gastric tumor	68	93	63	73	69
