# Supplementary material for: Highly sensitive MLH1 methylation analysis in blood identifies a cancer patient with low-level mosaic MLH1 epimutation
Source: Clin Epigenetics. 2019 Nov 28;11:171. doi: 10.1186/s13148-019-0762-6 (PMC6883525; doi:10.1186/s13148-019-0762-6)
Supplement: Supplementary file 8 — Additional file 8: Table S3. Variants identified in the mutational analysis of hereditary cancer genes in case 29. [file 13148_2019_762_MOESM8_ESM.pdf]

Table S3. Variants identified in the mutational analysis of hereditary cancer genes in case 29

Variant calling				Coverage		rs ID	MAF	*In silico* predictions					ClinVar Classification
								Splicing	Protein function				
Gene	Transcript	cDNA change	Predicted protein change	Allele Frequency (%)	Read Depth		genomAD (%)		SIFT	Mutation Taster	Polyphen2 /HumDiv	Polyphen2 /HumVar	
XRCC2	NM_005431.1	c.115G>A	p.V39M	45,19	104	rs730882040	0,01	No changes	Delete rious	Disease causing	Possibly damaging	Possibly damaging	Uncertain significance**
