# Supplementary material for: Highly sensitive MLH1 methylation analysis in blood identifies a cancer patient with low-level mosaic MLH1 epimutation
Source: Clin Epigenetics. 2019 Nov 28;11:171. doi: 10.1186/s13148-019-0762-6 (PMC6883525; doi:10.1186/s13148-019-0762-6)
Supplement: Supplementary file 9 — Additional file 9: Figure S5. Analysis of structural aberrations in case 29. A) Genome-wide SNP array profiling of blood DNA from case 29 is shown as Circos plots. Circos plot was divided into three concentric circles. Chromosomes are represented at the external circle with their centromeres painted in red. In the middle circle, external allelic peaks mark homozygous SNPs and internal allelic peaks heterozygous ones. Internal circle tracks log2 copy number lane: middle points indicate diploid genomic material; upper points, gains of genomic material and lower points, losses. Patient 29 displayed a diploid pattern throughout her genome without signs of loss-of-heterozygosity. B) CNV analysis in the MLH1 region of patient 29 by custom CGH array. Genes located in the analyzed region are represented at the bottom of the figure. Probes are displayed as green dots in a log2 graph. Gains and losses of genetic material are considered when more than five consecutive probes reach values of 2 or − 2, respectively. No CNV abnormalities were identified. [file 13148_2019_762_MOESM9_ESM.pdf]

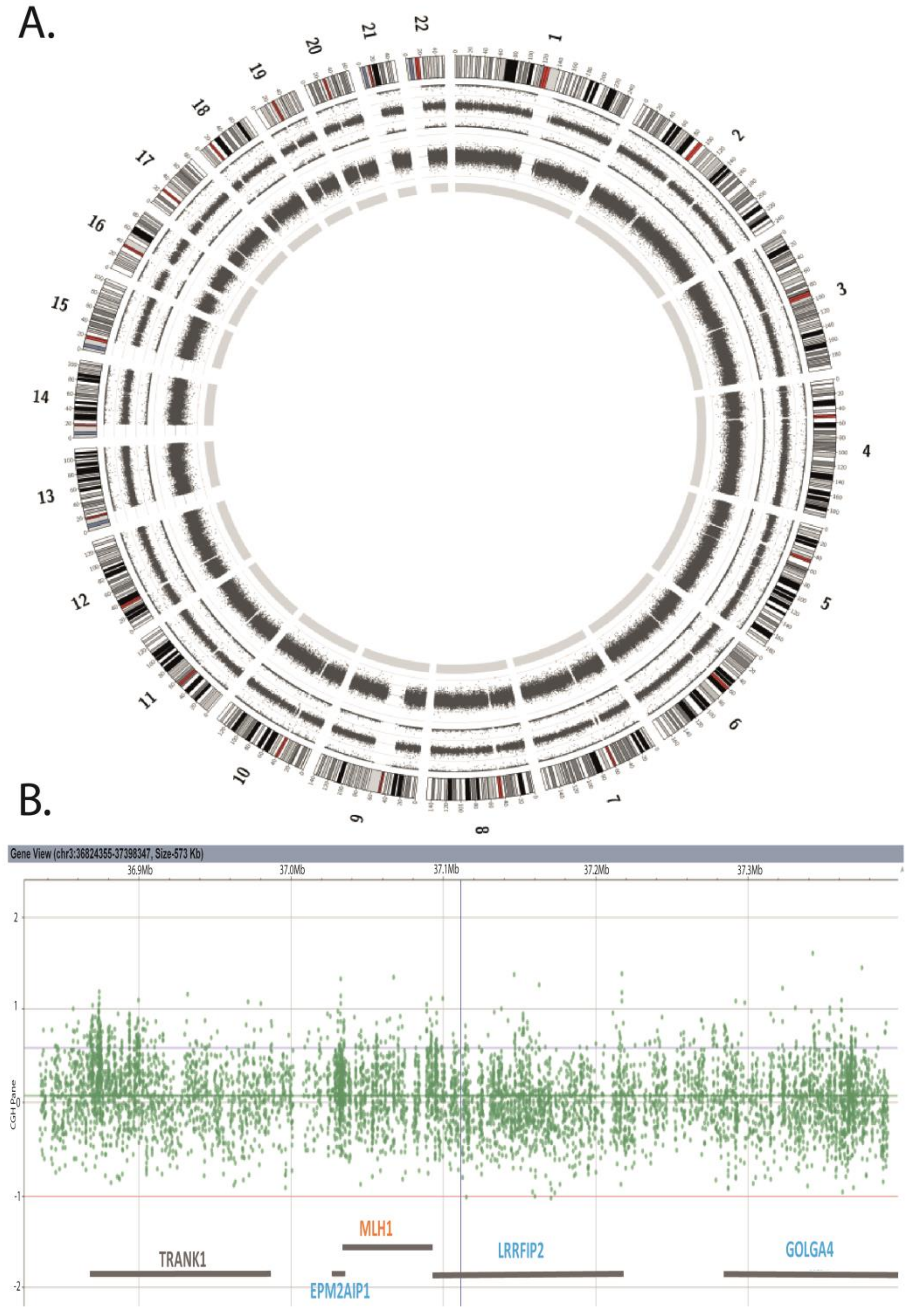

Figure S5. Analysis of structural aberrations in case 29. A) Genome-wide SNP array profiling of blood DNA from case 29 is shown as Circos plots. Circos plot was divided into three concentric circles. Chromosomes are represented at the external circle with their centromeres painted in red. In the middle circle, external allelic peaks mark homozygous SNPs and internal allelic peaks heterozygous ones. Internal circle tracks log2 copy number lane: middle points indicate diploid genomic material; upper points, gains of genomic material and lower points, losses.

Patient 29 displayed a diploid pattern throughout her genome without signs of loss-of-heterozygosity. **B)** CNV analysis in the *MLH1* region of patient 29 by custom CGH array. Genes located in the analyzed region are represented at the bottom of the figure. Probes are displayed as green dots in a log2 graph. Gains and losses of genetic material are considered when more than five consecutive probes reach values of 2 or -2, respectively. No CNV abnormalities were identified.
