# Supplementary material for: Highly sensitive MLH1 methylation analysis in blood identifies a cancer patient with low-level mosaic MLH1 epimutation
Source: Clin Epigenetics. 2019 Nov 28;11:171. doi: 10.1186/s13148-019-0762-6 (PMC6883525; doi:10.1186/s13148-019-0762-6)
Supplement: Supplementary file 10 — Additional file 10: Figure S6. Schematic representation of the methodological strategy and summary of the obtained results. [file 13148_2019_762_MOESM10_ESM.pdf]

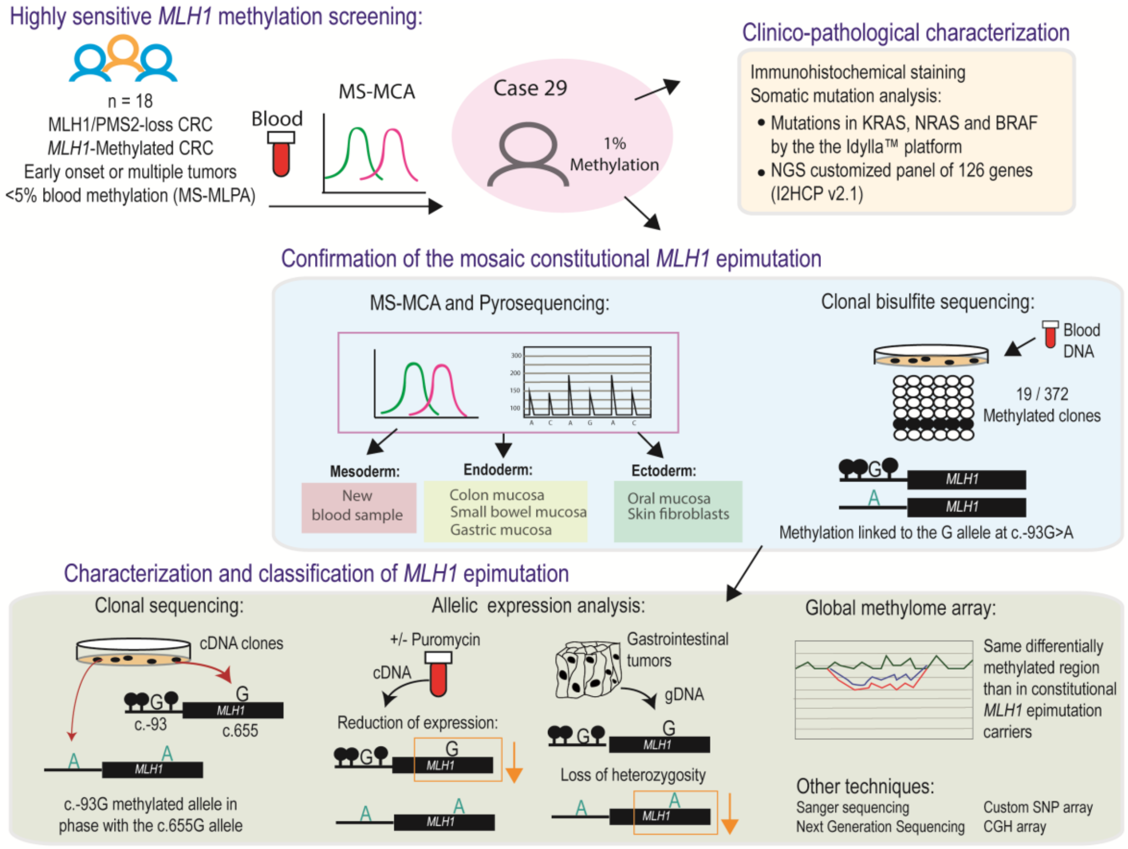

Figure S6. Schematic representation of the methodological strategy and summary of the obtained results
