# Supplementary material for: Highly sensitive MLH1 methylation analysis in blood identifies a cancer patient with low-level mosaic MLH1 epimutation
Source: Clin Epigenetics. 2019 Nov 28;11:171. doi: 10.1186/s13148-019-0762-6 (PMC6883525; doi:10.1186/s13148-019-0762-6)
Supplement: Supplementary file 11 — Additional file 11: Table S4. Reported patients with MLH1 epigenetic mosaicism at low proportion (≤10%). (*) According to the obtained results the three tumor lesions were clonally related. [file 13148_2019_762_MOESM11_ESM.pdf]

Table S4. Reported patients with *MLH1* epigenetic mosaicism at low proportion (≤10%). (*) According to the obtained results the three tumor lesions were clonally related.

Patient ID from the original publication	Cancer type (age of onset)	Methylation levels in blood	Detection technique	Confirmation by other tecniques	Analyzed in other tissues	Reference
29	CRC* (22), SBC* (24), GC* (25)	1-2%	MS-MCA	Clonal BS, Infinium 450k	BM, SF, CRM, SBM, GM	Pineda, Mur et al., 2012; current study
P	CRC (69)	2%	qMSP	Pyroseq, COBRA, Clonal BS		Ward et al. 2013
Q	CRC (39), skin (44)	3%	qMSP	Pyroseq, COBRA, Clonal BS		Ward et al. 2013
N	CRC (50, 52, 54)	3%	qMSP	Pyroseq, COBRA, Clonal BS		Ward et al. 2013
O	CRC (45)	4%	qMSP	Pyroseq, COBRA, Clonal BS		Ward et al. 2013
II-3	Asymptomatic (?)	4%	Pyroseq.	Clonal BS	saliva, hair folicles	Sloane et al. 2015
84-I	CRC (35)	8%	qMSP	Pyroseq, Clonal BS		Hitchins et al. 2011
G46	GC (60)	10%	Bisulfite seq	Clonal BS		Wu et al. 2012
