# Supplementary material for: Highly sensitive MLH1 methylation analysis in blood identifies a cancer patient with low-level mosaic MLH1 epimutation
Source: Clin Epigenetics. 2019 Nov 28;11:171. doi: 10.1186/s13148-019-0762-6 (PMC6883525; doi:10.1186/s13148-019-0762-6)

Table S1. Primers used in the study

A. Primers and conditions

Gene	Analysis	Primer name	Forward Primer (5'-3')	Reverse Primer (5'-3')	Ta (°C)
MLH1	MS-MCA	MLH1C_PCR_ext	TATTTTTGTTTTTATTGGTTGG	CCAATCAAATTTCTCAACTCTATA	50
		MLH1C_PCR_int	TGTTTTTATTGGTTGGATATTT	CCAATCAAATTTCTCAACTCTATA	50
		MLH1D_PCR_ext	AGGTATTGAGGTGATTGGTTG	CAATTCTCAATCATCTCTTTAATAACA	50
		MLH1D_PCR_int	GGTGATTGGTTGAAGGTATTTT	ATCATCTCTTTAATAACATTAACTAACC	50
	Pyrosequencing	Piro_PCR_MLH1_C	TTTYGGTATTTTTGTTTTTATTGGTTGG	[Btn]AAACAACTTAAATACCAATCAAATTTCTCAAC	58
		Piro_Seq_MLH1_C	TAAAAAYGAATTAATAGGAA	-	-
		Piro_PCR_MLH1_intron1	ATTTAGYGGTTAGTTAATGTTATTAAAGAGATG	[Btn]TAAACATACRCTATACATACCTCTACCC	56
		Piro_Seq_MLH1_intron1	AGTYGGGTTTATTTAAGGGTTA	-	-
	Clonal Bisulfite sequencing	PCR_MLH1_CD	TTTTAAAAAYGAATTAATAGGAAGAG	CAATTCTCAATCATCTCTTTAATAA	55
	Clonal Sequencing	PCR_MLH1_cDNA	GCATTCAAGCTGTCCAATCA	AGGTACAGGAATGGGTGTGTG	64
		Seq_MLH1_cDNA_int	GCAGTCCTTTGAGGATTTAGC	TCCATCAGCTGTTTTCGTT	-
	ASE (SNuPe)	rs179997_PCR_cDNA	CACAATGCAGGCATTAGTTTCTC	AGGTACAGGAATGGGTGTGTG	59
		rs179997_PCR_gDNA	GTTTCAGTCTCAGCCATGAG	ACACATGATTCACGCCACAG	55
		rs179997_snupe		TTCTCGACTAACAGCATTTCCAAAGA	50

B. Localization of the probes and regions analyzed in the study of *MLH1* methylation. The *EMP2AIP1-MLH1* CpG island (colored in dark purple) encompass the *MLH1* promoter and intron 1

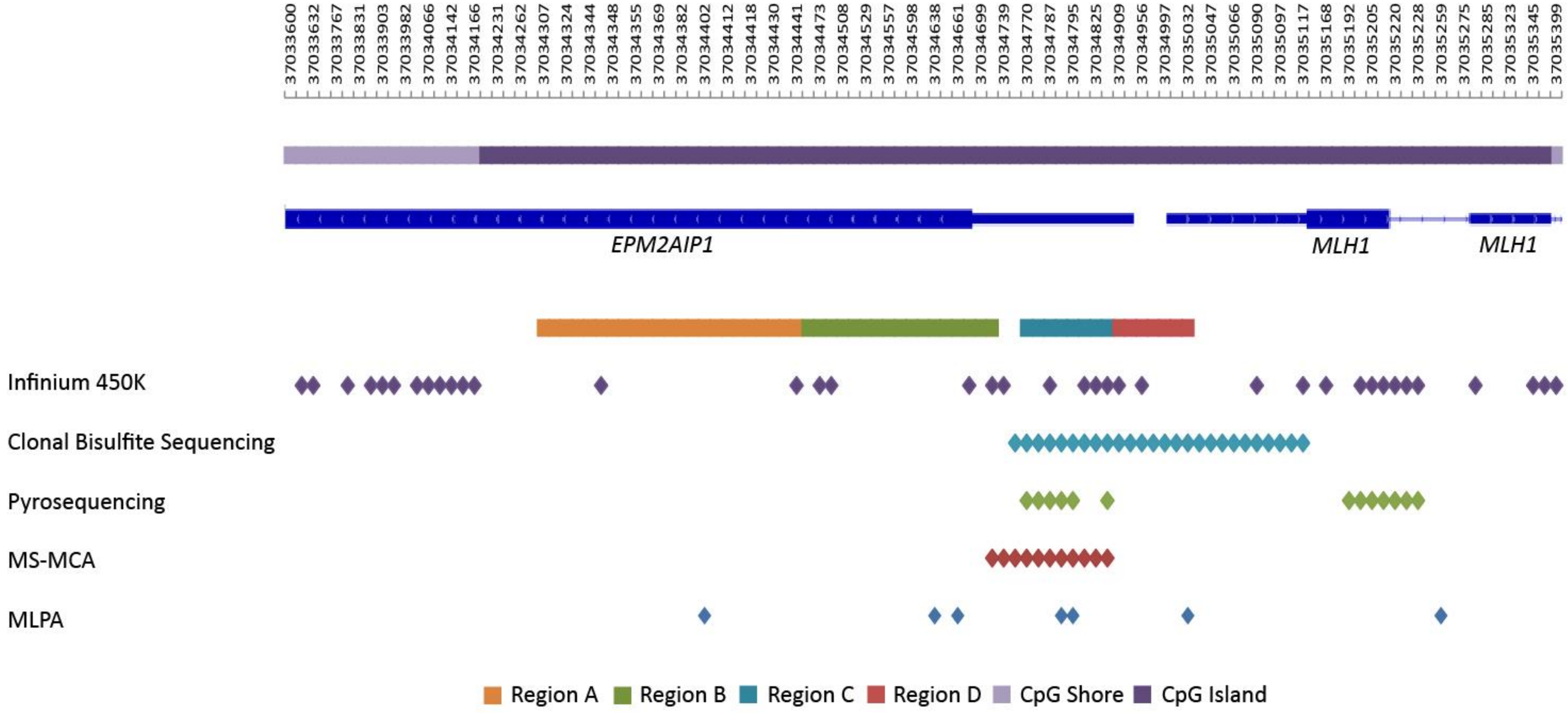

Supplement: Supplementary file 12 — Additional file 12: Table S5. A. Primers and conditions. B. Localization of the probes and regions analyzed in the study of MLH1 methylation. The EMP2AIP1-MLH1 CpG island (colored in dark purple) encompass the MLH1 promoter and intron 1. [file 13148_2019_762_MOESM12_ESM.pdf]
